# Lingual palpation for porcine cysticercosis: a rapid epidemiological tool for estimating prevalence and community risk in Africa

**DOI:** 10.1111/tmi.12760

**Published:** 2016-08-16

**Authors:** Helen L. Guyatt, Eric M. Fèvre

**Affiliations:** ^1^ Kimetrica LimitedNairobiKenya; ^2^ Institute of Infection and Global HealthUniversity of LiverpoolNestonUK; ^3^ International Livestock Research InstituteNairobiKenya

**Keywords:** *Taenia solium*, Cysticercosis, seroprevalence, tongue cyst prevalence, Epidemiology, control, surveillance, Africa, *Tænia solium*, cysticercose, seroprevalence, prévalence du kyste de la langue, épidémiologie, contrôle, surveillance, Afrique

## Abstract

**Objective:**

To assess the association between the prevalence of tongue cyst‐positive and antigen‐positive pigs across different settings in Africa, to evaluate whether examining pigs for cysts could be used as a rapid surveillance tool for identifying geographical areas with a higher probability of high transmission of cysticercosis.

**Methods:**

Published data were collated from 26 study sites across Africa that reported the prevalence of porcine cysticercosis by both lingual and serological examinations. The study sites were located in 10 countries across Africa.

**Results:**

Seroprevalence rates ranged from 4% to 41%. Despite the varied study sites, the relationship between the two variables was highly consistent and suggests identification of tongue cysts may be useful for cysticercosis surveillance. We found that all areas with more than 10% of pigs having cysts in their tongues had at least 30% seroprevalence (PPV of 100%), although this cut‐off is less reliable at predicting that an area is of low transmission (NPV of 84%).

**Conclusion:**

Assessing the prevalence of tongue cyst‐positive pigs is a potential rapid epidemiological tool for identifying areas at high risk of cysticercosis, although further refinement and validation is required using standardised data sets.

## Abstract (fr)

**Objectif:**

Evaluer l'association entre la prévalence de porcs positifs pour le kyste de la langue et les antigènes dans différents milieux en Afrique, évalué si l'examen des porcs pour les kystes pourrait être utilisé comme un outil de surveillance rapide pour identifier les zones géographiques avec une probabilité plus élevée de transmission élevée de la cysticercose.

**Méthodes:**

Les données publiées ont été collectées à partir de 26 sites d’étude à travers l'Afrique qui ont rapporté la prévalence de la cysticercose porcine à la fois par l'examen lingual et la sérologique. Les sites d’étude étaient situés dans 10 pays d'Afrique.

**Résultats:**

Les taux de séroprévalence variaient de 4% à 41%. Malgré les sites d’étude variés, la relation entre les deux variables était très cohérente et suggère que l'identification des kystes de la langue peut être utile pour la surveillance de la cysticercose. Nous avons constaté que toutes les zones avec plus de 10% des porcs ayant des kystes linguaux avaient au moins 30% de séroprévalence (PPV de 100%), quoique que ce seuil soit peu fiable pour prédire qu'une zone est de faible transmission (NPV de 84%).

**Conclusion:**

L’évaluation de la prévalence de porcs positifs pour le kyste la langue est un outil épidémiologique potentiel rapide pour identifier les zones à haut risque de cysticercose, bien que plus de raffinement et de validation soient nécessaires en utilisant des ensembles de données standardisés.

## Abstract (es)

**Objetivo:**

Evaluar la asociación entre la prevalencia de cerdos antígeno‐positivos y con quistes linguales a lo largo de diferentes lugares del África, para evaluar si el examinar a los cerdos en busca de quistes podría utilizarse como una herramienta rápida de vigilancia para identificar las áreas geográficas con una mayor probabilidad de alta transmisión de cisticercosis.

**Métodos:**

Se recopilaron datos publicados sobre 26 lugares de estudio a lo largo de África, en los que se había reportado la prevalencia de cisticercosis porcina tanto mediante examen lingual como serológico. Los lugares de estudio se localizaban en 10 países a lo largo de África.

**Resultados:**

Las tasas de sero‐prevalencia estaban entre un 4% y 41%. A pesar de la variedad de lugares de estudio, la relación entre las dos variables era altamente consistente y sugiere que la identificación de quistes linguales podría ser una herramienta útil para la vigilancia de la cisticercosis. Encontramos que en todas las áreas con más de un 10% de los cerdos con quistes en sus lenguas había una seroprevalencia de al menos un 30% (PPV de 100%), aunque este punto de corte es menos fiable a la hora de predecir áreas de baja transmisión (NPV de 84%).

**Conclusión:**

Evaluar la prevalencia de cerdos positivos para quistes linguales es una herramienta epidemiológica potencial para identificar áreas de alto riesgo de cisticercosis, aunque se requiere un mayor refinamiento y validación utilizando sets de datos estandarizados.

## Introduction

The Food and Agriculture Organization (FAO) has identified cysticercosis as the most important foodborne parasite globally 1 and WHO is now committed to rolling out control of this disease by 2020 2. The tools for control are or will soon be available on a large scale, including a pig vaccine, and oxfendazole treatment of pig cysticercosis. The challenge is in delivering these interventions. A key issue is identifying where in a country to target control efforts. It is well accepted that most pig‐rearing regions within a country will be at risk. However, within these, high‐ and low‐risk areas need to be identified, especially given the knowledge that intensity of infection can vary spatially 3. To undertake such large‐scale surveillance, a cheap, simple, and rapid tool for diagnosing community risk is needed. This could be similar to those that have been established and implemented for schistosomiasis and the soil‐transmitted nematodes 4, where large volumes of data on low‐ and high‐risk communities can be rapidly generated and used in targeting interventions at appropriate geographical areas.

Lingual palpation (or tongue inspection) for cysts is a practice carried out by pig producers, buyers and veterinarians alike to rapidly screen pigs for cysticercosis. However, because it has been shown to have low sensitivity in identifying infected animals (as low as 21%) 5, it has been discarded as a potential diagnostic tool for defining individual infection in favour of more sensitive serological tests. As tongue cysts are more likely to be observed in heavily infected pigs 6, the prevalence of pigs positive for tongue cysts could reflect the prevalence of pigs with heavy infection. Given the obvious cost, resource, and time advantages of lingual palpation over serological examinations 7, we believe that the potential of this tool at the population level in identifying community risk should be explored. The objective would be to use this simple test to survey across pig populations and rapidly identify infected communities where the cost‐effectiveness of intervening would be high because of the high intensity of transmission.

## Methods

We undertook a comprehensive literature review of published community studies in Africa that collected both serological (presence of circulating antigen using a monoclonal antibody‐based sandwich enzyme‐linked immunosorbent assay [Ag‐ELISA]) and lingual palpation data for pigs (through physical examination of the ventral surface of the tongue for cysts). Only data from household surveys (as opposed to markets or slaughter houses) were included.

In total, we identified 26 study sites across 10 countries in Africa (Cameroon 8, 9, 10, 11, Chad 8, DRC 12, Kenya 13, 14, Mozambique 15, Senegal 16, South Africa 17, 18, Tanzania 19, The Gambia 16 and Zambia 20, 21) conducted between 1999 and 2010 (see Table 1). Apart from the two studies in Kenya, which used the HP10 antigen test as described by Harrison *et al*. 22, all studies assessed the presence of the B158/B60 antigen in the serological examinations using the approach developed by Dorny *et al*. 5, 23, occasionally with slight modifications. The majority of countries were represented by 1 or 2 study sites, the exceptions being Cameroon and Zambia, which together accounted for half of all sites. Between 93 and 452 pigs were examined per study. Most studies represented samples from groups of villages targeting pig‐keeping households, although one study targeted cattle‐keeping households with data on just 93 pigs from 416 households sampled in 164 sublocations 14. In most cases, the same pigs were examined in both tests, or only a subsample was assessed for serology 8, 13, or tongue inspection 10, 12.

**Table 1:** Prevalence of serological and clinical porcine cysticercosis from same settings across Africa

Country	Location	Sample	Year of survey	Antigen prevalence, %	Tongue cyst prevalence, %	Reference
Cameroon	Mayo‐Danay (Northern)	441 pigs (139)*	1999	38.9	15.4	8
	Bafou (Western)	400 pigs (15 villages)	2000	9	5.5	9
	Bamendou (Western)	307 pigs (12 villages)	2000	13.7	6.8	9
	Batibo (North West)	271 pigs (192)† (2 villages)	2001	27.7	0.5	10
	Bafut (North West)	214 pigs	Not given	4.2	2.8	11
	Santa (North West)	285 pigs	Not given	10.2	4.2	11
Chad	Mayo‐Kebbi (South West)	411 pigs (125)*	1999	40.8	26	8
DRC	Bas‐Congo	153 pigs (145)† (5 villages)	2009	41.2	5.5	12
Kenya	Homa Bay (Western)	392 pigs (232)* (42 villages)	2010	32.8	5.6	13
	Western and Nyanza	93 pigs (164 sublocations)	2010–2012	17.2	9.7	14
Mozambique	Doume, Angonia (North West)	383 pigs (6 villages)	2007	34.2	13.1	15
	Uloungue, Angonia (North West)	278 pigs (5 villages)	2007	36.0	12.2	15
Senegal	Bignona (Southern)	433 pigs (15 villages)	2007–2008	8.9	1.0	16
	Kolda (Southern)	449 pigs (17 villages)	2007–2008	13.2	0.1	16
	Ziguinchor (Southern)	452 pigs (16 villages)	2007–2008	6.4	0.3	16
South Africa	Eastern Cape	261 pigs (21 villages)	2003	40.6	11.9	17, 18
Tanzania	Mbeye	300 pigs (15 villages)	2007–2008	30.7	6	19
	Mbozi	300 pigs (15 villages)	2007–2008	32.0	11.7	19
The Gambia	Western	371 pigs (15 villages)	2007–2008	4.8	0.2	16
Zambia	Kalomo (Southern)	98 pigs (21 villages)	2000	20.8	8.2	20
	Sinda (Eastern)	151 pigs (3 villages)	2001	9.3	5.2	20
	Gwembe (Southern)	385 pigs	2002–2003	34	21.6	21
	Monze (Southern)	387 pigs	2002–2003	22.7	8.8	21
	Petauka (Eastern)	384 pigs	2002–2003	14.6	6.5	21
	Katete (Eastern)	385 pigs	2002–2003	19.2	7.5	21
	Mongu (Western)	150 pigs	2002–2003	30	7.3	21

In some cases, a smaller number of pigs were examined for the serological test* or lingual palpation^†^.

The sampling framework, if given, varied markedly across studies at each level (village, household and pigs within households). For instance, in some studies villages were purposively selected based on certain risk factors such as large numbers of pigs, accessibility and/or willingness to participate 12, 17, 20, whereas others were selected at random 13, 19, or at random within defined strata such as levels of pig management 16. Pig‐keeping households within these villages were sampled collectively 10, 12, 13, at random 16, 19, or using snowballing techniques 15, 21. In most cases, all eligible pigs in a selected household were examined, although some studies reported random sampling of subgroups of pigs if numbers in a household exceeded two 13, 19 or four 21.

## Results

**Figure 1:**
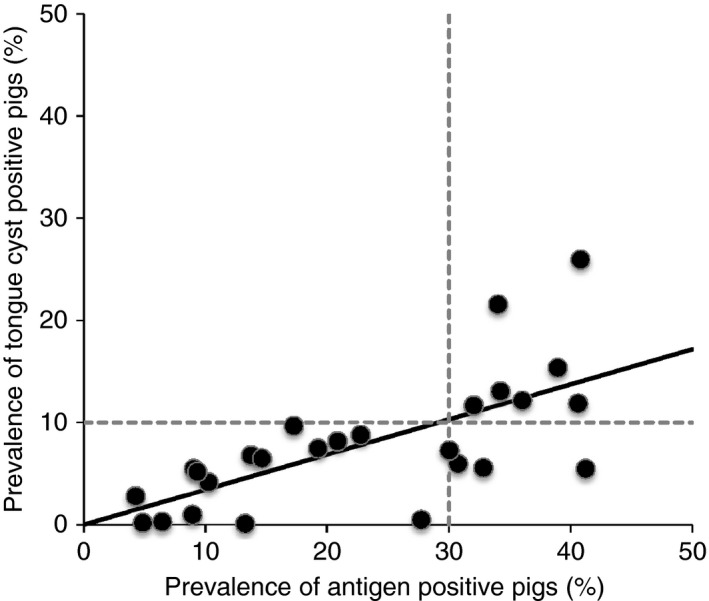
The relationship between the porcine cysticercosis markers of antigen‐positive prevalence and tongue cyst‐positive prevalence from 10 African countries. The circles represent a given study site which collected data on both the proportion of pigs with a positive response to a serological Ag‐ELISA and the proportion with one or more tongue cysts in clinical examination. A total of 26 study sites are represented (see Table 1 for data sources). The horizontal dashed line represents a tongue cyst prevalence of 10%, and the vertical dashed line represents an antigen‐positive prevalence of 30%. The solid black line represents the best fit regression with a coefficient of 0.3434 and an *R*‐squared of 0.793 (*P* < 0.001).

Figure 1 shows the relationship between the prevalence of cysticercosis as assessed serologically (antigen‐positive pigs) and that assessed by lingual palpation (tongue cyst‐positive pigs). Despite the wide range of study sites and sampling frameworks represented in this Figure, the relationship is highly consistent. Furthermore, a linear regression model indicated an R‐squared of 0.79, and a coefficient of 0.34 (95% CI 0.27–0.42) suggesting a strong correlation between the two variables.

In all studies, the prevalence of tongue cyst‐positive pigs was less than the prevalence of antigen‐positive pigs, an expected outcome given the known sensitivity issues of lingual examination. However, given the data available at present, if the prevalence of tongue cyst‐positive pigs is >10%, then the serological prevalence appears to always be >30%. Tongue cyst‐positive rates >10% varied between 12% and 26%, and suggest that a prevalence >10% would indicate a seroprevalence in pigs of more than 30%, with a positive predictive value (PPV) of 100% (7/7) (i.e. only true positives are identified). If 10% or less of pigs have cysts on their tongues, seroprevalence can vary widely between 4% and 41%, and lingual examination becomes a far less valuable indicator of intensity of infection in the population. From the data available, it seems that this cut‐off would identify seroprevalence rates of 30% or less with a negative predictive value (NPV) of 84% (16/19) (i.e. a sixth of those sites identified as having this low seroprevalence would actually have a high prevalence). In fact, of the 10 studies with a seroprevalence >30%, three had a tongue cyst prevalence below 10%, although two of these had seroprevalences close to the cut‐off (30.7 and 32.8).

## Discussion

If, as we suggest, the prevalence of pigs with tongue cysts does relate to the prevalence of heavy infection, then its relationship with the prevalence of infection (as assessed by serology) would be predicted to be nonlinear if the distribution of cysts in a pig population is overdispersed (i.e. most pigs have a few cysts and a few pigs have many cysts). These overdispersed distributions in parasite numbers have been observed and quantified for other helminth infections such as *Ascaris lumbricoides* 24, and it is conceivable that the relationship between prevalence of tongue cyst‐positive pigs and the prevalence of antigen‐positive pigs can be quantified using the negative binomial k 24. This would allow a formula for predicting the equivalent antigen‐positive prevalence given a defined tongue cyst‐positive prevalence. Fitting the negative binomial to cyst numbers in an experimentally infected pig population suggested k values in the region of 0.23–0.37 7, although similar data from naturally infected pig populations are scarce.

The data presented here on lingual and seroprevalence in same settings represent published studies in the international scientific literature to date in Africa. Although there are concerns related to the specificity of the antigen test in diagnosing *T. solium* infection 5, given the cross‐reactivity with the metacestode stage of *T. hydatigena*, this appears to be more a problem in parts of Asia, such as Vietnam 25, where the prevalence of *T. hydatigena* in pigs can reach levels in excess of 60%. This contrasts markedly with studies in Africa where *T. hydatigena* in pigs rarely exceeds a few per cent 26, 27, 28. It appears that although *T. hydatigena* can be found at high prevalences in goats in Africa, the prevalence in pigs is consistently low, in the range of 2 to 6% (P. Dorny, pers. comm.). Although both *T. solium* and *T. hydatigena* can be a consequence of bad sanitation and slaughter practices, their transmission and risk factors can be quite different, and as a result, we may not see them at equivalent prevalences in pig populations.

Indeed, the consistency of the relationship we observe between lingual prevalence and seroprevalence suggests that any modifying effects the presence of *T. hydatigena* does have are either extremely small or consistent across all settings. However, we would call for further data to validate this relationship, particularly in areas of intense transmission (there were no studies with a seroprevalence in excess of 41%). There are likely to be unpublished data within countries as part of ongoing surveillance or research programmes which could complement this data set. Furthermore, the collection of such data should be encouraged in future activities. Data are required from village‐based field surveys, not surveys undertaken in slaughterhouses or markets where pigs may already have been pre‐selected to be tongue cyst free.

The data also need to be collected using standardised sampling frameworks. Various approaches to sampling villages, households and pigs were employed in the published data presented here. Studies that only sample a few households or pigs in a whole village are unlikely to be representative unless villages are highly homogenous in their levels of cysticercosis. We propose a multicountry approach in which villages with a range of pig population sizes and expected prevalences are comprehensively sampled and examined both clinically and serologically. This would help establish whether the NPV of 84% observed using this limited data set (with the possible concerns that a proportion of high‐risk areas could be missed) is still applicable when the data are standardised. Such an activity could form part of the global network for the elimination of cysticercosis proposed by WHO 2 and contribute to validating a rapid epidemiological assessment tool much needed in implementing these programmes. This would also be an opportunity to evaluate what is meant by a high‐risk area for cysticercosis. We applied a threshold of 30% porcine seroprevalence consistent with previous publications that refer to values of between 30% and 60% as reflecting areas with a high prevalence or high endemicity 13, 18, 29. A review of the relationship between porcine seroprevalence and human morbidity would be useful in establishing a standard for defining a high‐risk area for cysticercosis.

## Conclusion

The data presented suggest that if more than 10% of pigs are tongue cyst‐positive in an area, then these areas will be at high risk of cysticercosis with more than 30% of pigs being infected. Although there is a potential for 16% of high‐risk areas to be missed using this 10% lingual prevalence cut‐off, further data and quantification of the relationship between the proportion of pigs with cysts in their tongues and antigen‐positive pigs could provide a cheap and rapid decision‐support tool for identifying target areas for cysticercosis control, and in evaluating and monitoring control efforts.
